# Longterm efficacy of a patient focused intervention in patients with asplenia– a three year follow-up of the PrePPS trial

**DOI:** 10.1007/s15010-025-02472-5

**Published:** 2025-01-29

**Authors:** Johannes Camp, Valerie Heine, Marianne Bayrhuber, Natascha Anka, Manuela Glattacker, Erik Farin-Glattacker, Siegbert Rieg

**Affiliations:** 1 Division of Infectious Diseases, Department of Medicine II, Medical Centre, Faculty of Medicine, University of Freiburg, University of Freiburg, Hugstetter Str. 55, 79106 Freiburg, Germany; 2 Faculty of Medicine, Section of Health Care Research and Rehabilitation Research, University of Freiburg, Freiburg, Germany

**Keywords:** Asplenia, Vaccinations, Vaccines, HAPA, Post-splenectomy sepsis

## Abstract

**Objectives:**

This study aimed to reassess the long-term impact of a Health Action Process Approach (HAPA)-informed intervention on guideline adherence among asplenic patients and their physicians, three years post-intervention.

**Methods:**

This follow-up study was conducted within the framework of the interventional PrePSS (Prevention of Postsplenectomy Sepsis Score) study. Patients aged 18 or older with anatomical asplenia were in enrolled in a prospective controlled, two-armed historical control group design. The intervention group received tailored educational materials, medical alert cards, and a telephonic HAPA-based intervention. A telephonic follow-up assessment was conducted three years post-intervention, evaluating adherence to preventive measures using the PrePSS-score (range 0–10) and propensity-score based overlap weighting.

**Results:**

Out of 106 patients who had received the intervention, 79 (75%) completed the three-year follow-up. The PrePSS-scores further increased compared to six months post-intervention (median of 8.08 points vs 7.60 points), with notable improvements in vaccination coverage and availability of emergency antibiotics. Only four participants reported severe infections requiring hospitalization, none of which were typical of post-splenectomy sepsis. 47/79 (59%) of participants contracted SARS-CoV2 but clinical courses were mild with only one patient needing hospital and ICU treatment.

**Conclusions:**

Patients who had received our novel telephonic intervention exhibited significant and sustained improvement in adherence to guideline-based preventive measures at three years post intervention. HAPA-based interventions may yield more sustained effects than traditional nudging strategies.

## Introduction

Asplenia or reduced splenic function increases susceptibility to severe infections like post-splenectomy sepsis (PSS) [10, 18, 20, 21]. Despite available preventive measures outlined in international guidelines, adherence remains low, partly due to gaps in patient education [7]. Efforts to improve adherence, such as spleen registries and specialized services, face challenges like infrastructure limitations and communication issues [12], [22]. Recently, we proposed an intervention informed by the Health Action Process Approach (HAPA) theory aimed at enhancing guideline adherence among asplenic patients and their physicians [8]. While we found a significant increase in adherence to recommended measures at six months after the intervention (mean PrePSS-scores of 3.73 and 7.70 in control and intervention group, respectively), our goal was to achieve a sustained long-term effect. Therefore, the present study aimed to reassess this outcome at three years post intervention.

## Methods

**Fig. 1:**
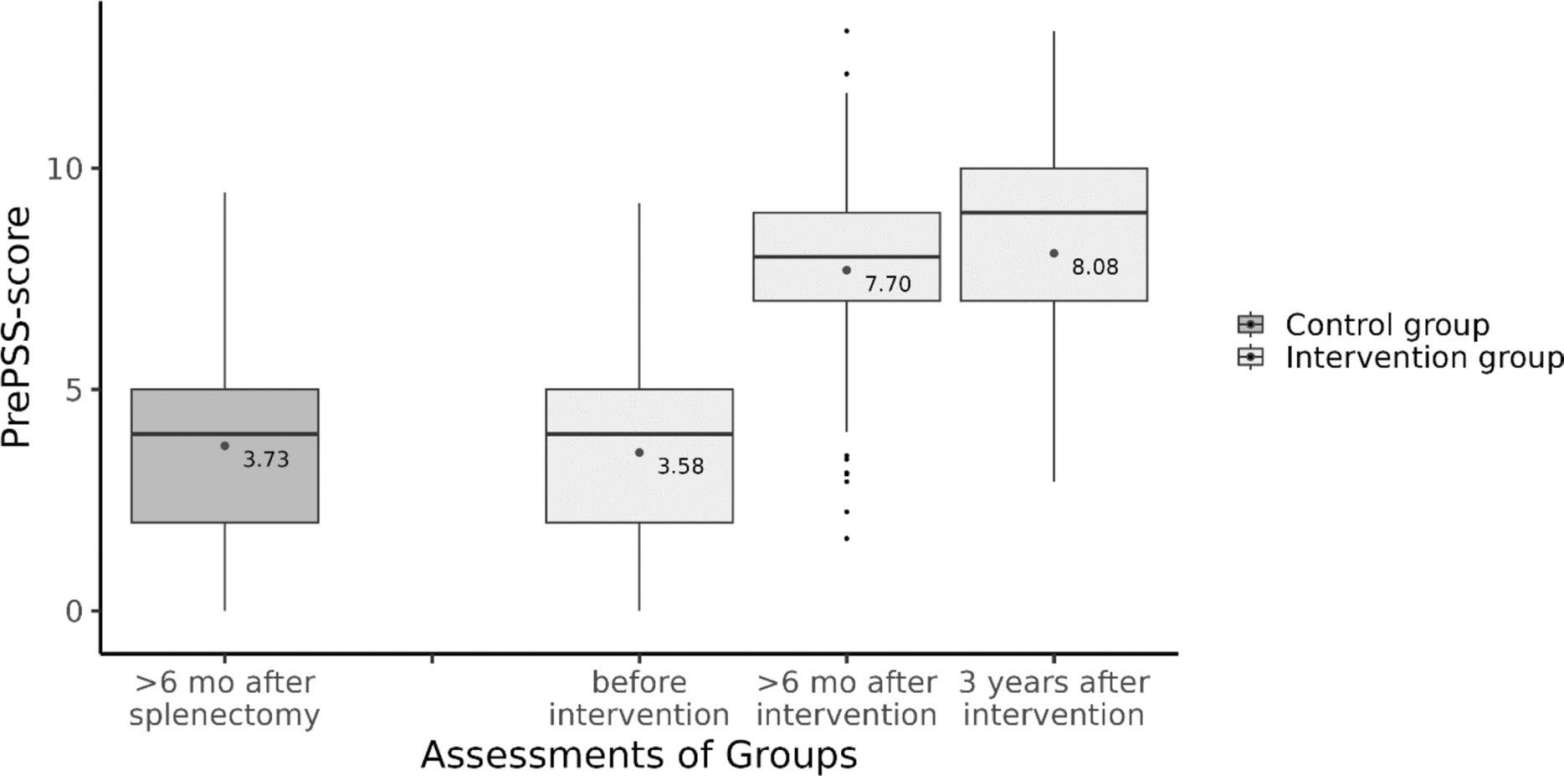
PrePPS-score in the historical control group and the intervention group before, > 6 months and three years after the intervention. Boxplots show median (black line) and interquartile range (box). Whiskers extend to ± 1.5 * interquartile range. Small black dots represent outliers, medium grey dots represent the mean PrePSS-score as given in the annotation

This study was conducted as part of a planned follow-up within the scope of the PrePPS study [1]. An in depth discussion of the intervention has been published elsewhere [6] as have been the results of the evaluation regarding the primary and secondary outcomes [1, 5]. Briefly, patients aged 18 years or older, with anatomic asplenia were eligible to be enrolled in our study using a prospective controlled, two-armed historical control group design with baseline, post- and follow-up measurement. Patients of the control group underwent splenectomy at least sixth month prior to study inclusion. Participants for the intervention group were prospectively enrolled between February 2019 and January 2021. The study was conducted as a single center study at the University Medical Center Freiburg in cooperation with the insurance provider AOK Baden-Wuerttemberg, Germany. Patients in the intervention group were sent tailored educational materials and a medical alert card for patients with asplenia and received a novel, telephonic, manual-based, individual intervention (T0) following the HAPA theory, combining an information-giving section and intervention components that promote motivation for initiation and planning of recommended infection prevention measures (i.e. vaccinations, medical alert card, emergency antibiotics) [9], [24], [3, 13]. Sixth months and again three years after the intervention patients in the intervention group were followed-up via telephone call (T1 and T2-FU) and the implementation of the recommended prevention measures was assessed using the PrePSS-score. Additional documents (e.g. discharge letters in case of hospitalisation) were obtained where necessary either via the patients themselves or their general practitioners. All data were entered into an electronic database system (REDcap). Here we report on the data of the three years follow-up (T2-FU) (Fig. 1).

### Funding source

**Fig. 2:**
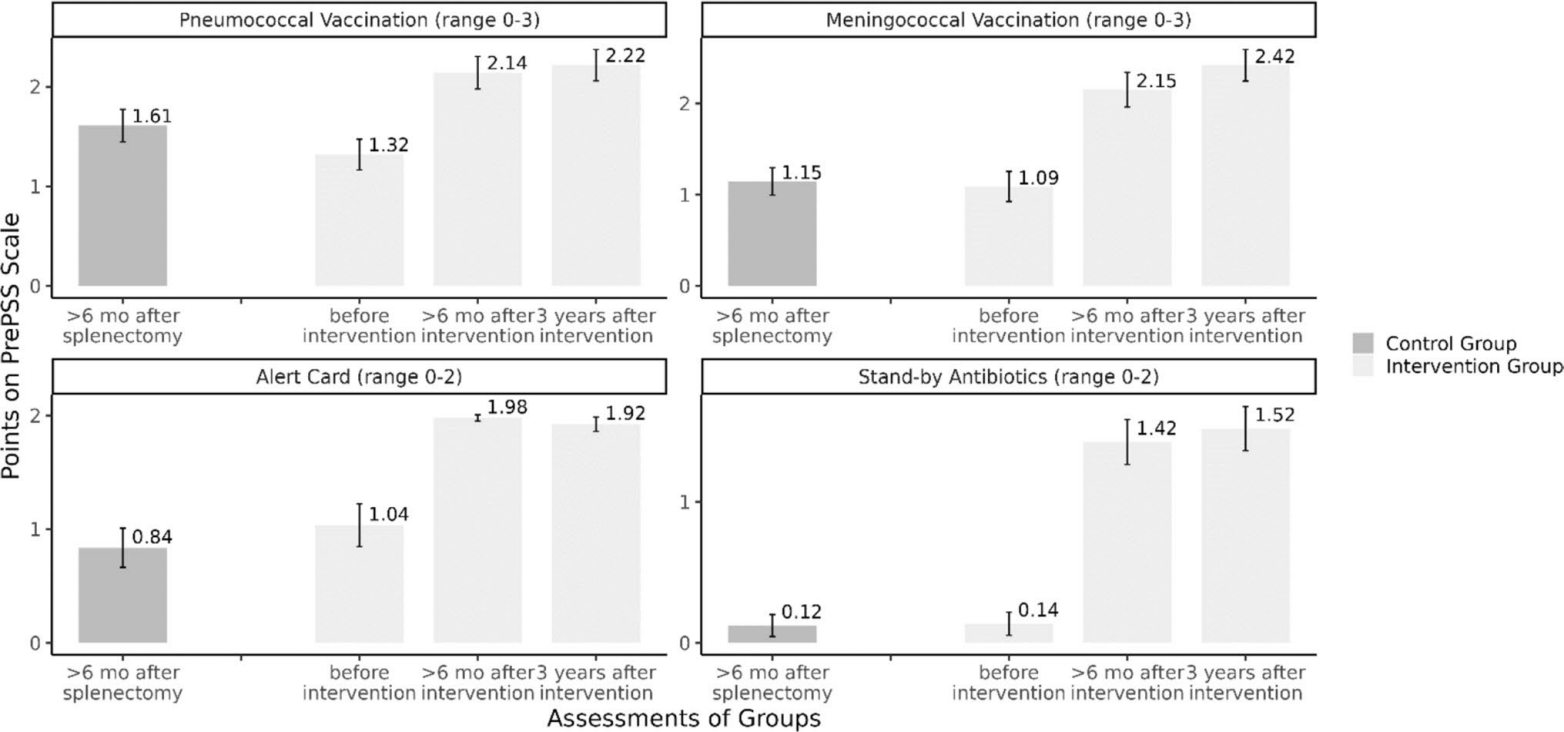
Mean points for different items of the PrePSS-score in the historical control group and the intervention group before, > 6 months and three years after the intervention

The study is funded partially by Innovationsausschuss of the Gemeinsamer Bundesausschuss, Wegelystraße 8, 10623 Berlin (grant number: 01VSF17049). The funding body was not involved in any aspect of the design of the study, collection of study data, in writing the manuscript, or in the decision to submit this article for publication (Fig. 2).

### Outcomes

Primary outcome of the follow-up interview was the adherence to preventive measures in the intervention group three years after the intervention compared to six months after the intervention. Adherence was measured by the study-specific PrePSS-score (range 0–10 points), which has been described previously [5], [1]. Briefly, it assesses the following items: (a) receipt of guideline-conform sequential pneumococcal vaccination (0–3 points), (b) guideline-conform meningococcal vaccinations (0–3 points), (c) prescription and availability of stand-by antibiotics for emergency treatment (0–2 points) and (d) handing out of and carrying a medical alert card (0–2 points). Secondary outcomes included history of serious infection and/or hospitalization particularly related to COVID-19.

### Statistical analysis

Categorical variables are given as absolute and relative frequencies, continuous variables as means with standard deviation or medians with the first and third quartile. We adjusted for possible residual confounding due to missing randomization by using overlap weights, which is a propensity score weighting method [17]. We estimated the propensity score (PS) using a logistic regression model including age, sex, socio-economic status, cause of splenectomy, subjective disease knowledge, Charlson comorbiditiy index and intake of immunosuppressive agents as main effects. Overlap weights were then generated by assigning treated individuals the weight 1-PS, while those in the control group were assigned the weight PS. The average treatment effect in the weighted samples was estimated using a generalized linear model and confidence intervals were calculated using bootstrapping since the robust variance estimator is biased for propensity score weighting methods [11]. All estimated parameters are presented with their 95% confidence intervals. The whole analysis was conducted using R version 4.1.2 (Table 1).

**Table 1:** Outcome parameters in the intervention group 3 years after the intervention

Variable	Intervention group	
	N/M	%
Status		
Alive	79/106	74,5
Deceased	18/106	17,0
Due to underlying disease	11/18	61,1
Unknown causes	7/18	38,9
LTFU	9/106	8,5
Severe Infection (necessitating hospitalization)	5/79	6,3
Number of times medical alert card was used		
0	47/79	59,5
1	6/79	7,6
> 2	16/79	20,3
Number of times stand-by antibiotics were used		
0	63/79	79,7
1	10/79	12,7
2	6/79	7,6
Number of PCR confirmed COVID cases		
0	47/79	59,4
1	28/79	35,4
2	4/79	5,1
Treated as outpatients	31/32	96,9
Necessitated hospitalization	1/32	3,1
Admission to ICU	1/32	3,1

### Ethical consideration

The study and intervention were approved by the ethics committee of the Albert-Ludwigs-University, Freiburg, Germany (vote no. 380/18). We followed the ethical standards set by the Helsinki Declaration of 1975, as revised in 2004. The PrePSS study is registered in the German Clinical Trials registry (DRKS00015238).

## Results

In total, 79/106 (75%) of patients who had received the intervention (intervention group) completed the 3 year follow-up (T2). Median age of patients was 64 years [58–74] and 63/106 (59%) were female. 9/106 (8.5%) patients were lost to follow up and 18/106 (17%) had died in the meantime. In the majority of cases, the underlying disease (mostly malignoma) was determined as the cause of death, yet in 7/18 (39%) deceased patients the cause of death could not be ascertained (Table 2).

**Table 2:** Epidemiology and clinical characteristics of asplenic patients hospitalized for infections

Age	Sex	PrePPS Score	Infection
60	F	9	Bacterial pneumonia, causative agent not identified
62	M	9	Sepsis associated with postoperative pancreatic abscess, S. constellatus and F. nucleatum recovered
80	M	9	Aortic valve endocarditis, causative agent not identified
33	F	9	Tonsillitis (two episodes)
61	M	10	COVID-19, necessitating ICU treatment and ventilation

^*^All patients survived the respective infections detailed in this table

In order to enable meaningful comparisons with the control group and the previous timepoints of the intervention group we applied overlap weights to balance for residual confounding (Table S1). At the T2 we found that PrePSS scores in the intervention group had again increased compared to T1 (median of 8.08 points vs 7.60 points). The strongest increase was seen in meningococcal vaccinations with 65% having complete coverage compared to 49% at T1 (increase by 16%). Availability of emergency antibiotics also increased by 8% in total. While pneumococcal coverage saw only a moderate increase (increase by 2.4%), the availability of the medical alert card slightly decreased (3.8%), because participants had lost their card and had not applied for a replacement. Overall, 27.6 (27%) of patients achieved the full PrePPS score of 10 points at T2, compared to 21.4 (21%) patients at T1 (Table 3).

**Table 3:** Scores of individual PrePSS-score items stratified by group

Variable	Control group (n = 109.3)		Intervention group T1 (n = 102.8)		Intervention group T2-FU (n = 102.8)	
	N	N	N	P	N	P
Guideline-conform sequential pneumococcal vaccination^1^						
3 Points	24.5	22,4%	43,4	42.2%	45.9	44.6%
2 Points	21.0	19,3%	31,7	30.8%	34.4	33.5%
1 Point	60.4	55,3%	26,6	25.9%	21.4	20.8%
0 Points	3.3	3,0%	1,1	1.1%	1.1	1.1%
Guideline-conform meningococcal vaccination^2^						
3 Points	8.2	7.5%	50.1	48.7%	66.7	64.8%
2 Points	19.6	17.9%	26.4	25.7%	16.8	16.3%
1 Point	61.5	56.2%	18.1	17.6%	14.9	14.5%
0 Points	20.0	18.3%	8.2	7.9%	4.4	4.3%
Handing-over and carrying a medical alert card						
2 Points	38,3	35.0%	100.7	97.9%	96,8	94.1%
1 Point	14.9	13.6%	2.1	2.1%	4.1	4.0%
0 Points	56.1	51.4%	0.0	0.0%	1.9	1.9%
Stand by-antibiotic prescribed and available (‘pill in the pocket ‘)						
2 Points	3.4	3.1%	65.6	63.8%	73.9	71.9%
1 Point	6.6	6.0%	15.2	14.8%	8.4	8.2%
0 Points	99.3	90.8%	22.0	21.4%	20.5	19.9%

^1^13-valent conjugate vaccine PCV-13 (Prevenar-13^®^) after ≥ 2 months followed by 23-valent polysaccharide vaccine PSV-23 (Pneumovax^®^)

^2^Tetravalent meningococcal conjugate vaccination Men-ACWY (Menveo^®^, Nimenrix^®^), two doses at least two months apart; meningococcal serotype B vaccine Men-B (Bexsero^®^ [two doses] or Trumenba^®^ [three doses])

Four of 79 participants who completed T2-FU reported severe infections necessitating in-hospital treatment. No pathogens that are typically associated with PSS were recovered, however, in one case of bacterial pneumonia the causative pathogen could not be determined. Pathogens that were identified were not covered by the recommended vaccinations and indeed no vaccinations are or were available against these organisms at that particular time point.

Most of the patients never had to use their medical alert card or their emergency antibiotics. However, 22/79 (28%) patients showed their medical alert card to medical professionals at least once and 16/79 (20%) used their emergency antibiotics on at least one occasion.

We also obtained data on COVID-19 infections at T2-FU. Overall, 47/79 (59%) of participants had PCR-evidence of infection with SARS-CoV2. There were additional cases in which only rapid antigen testing was performed (mainly in the later stages of the pandemic), which were not counted for the analysis. Outcome was favorable with only one patient needing hospital and ICU treatment. This case occurred in late 2020, when vaccinations against SARS-CoV2 were not yet available.

## Discussion

Following up on our prospective controlled cohort, we were able to demonstrate that a novel telephonic HAPA-based intervention led to sustained improvements in adherence to recommended preventive measures in asplenic patients. Three years after the intervention overall PrePPS scores increased as did individual vaccination coverage.

We observed a comparatively high follow-up completion rate, with 79 out of 88 surviving patients (90%) successfully followed up after three years. The relevant number of deaths (18/106, 17%) is partly to be expected because splenectomy is increasingly performed for non-traumatic reasons with the main reason being a malignant disease [16], [10]. In our cohort, the percentage of patients suffering from malignoma was even higher than in the literature with more than half of the patients undergoing splenectomy for hematooncological reasons. Prognosis in such a cohort is limited compared to studies analyzing patients after traumatic asplenia [14]. Additionally, it is highly likely that our data underestimate underlying disease as cause of death since multiple patients who failed to respond to our efforts or who were confirmed to be dead by next of kin were known to suffer from advanced malignoma since before the inclusion in the study.

The long-term effects of telephonic interventions are subject to ongoing debate and seem to depend heavily on the actual intervention. A review by Tonapa and colleagues found, e.g., that telecoaching in patients with heart failure improved self-care behaviour and quality of life. However, follow-up times were quite short (range 3–9 months) and longterm effects thus not studied [15]. On the other hand two RCTs studying the effect of a digital intervention on depressive symptoms in diabetic patients found that albeit there was a significant improvement three months after the interventions, these effects had vanished after six months of follow-up [2]. Following our intervention, however, according to the PrePSS score preventive measures were upheld for or even re-newed after three years (e.g. seeking recommended re-vaccinations, renewing antibiotics prescriptions). The robustness of this effect may in part be due to the high degree of patient involvement and empowerment using a HAPA based methodology. The efficacy of this intervention is possibly even greater because of the nature of most of the recommended preventive actions. Vaccinations, e.g., once administered have by design a longterm effect. However, even the item most prone to degradation (i.e. the availability of the medical alert card) showed only minor loss rates. Additionally, we speculate that the promulgation of asplenia related information and distribution of medical alert cards via general practitioners might lead to secondary preventive effects in other patients with asplenia. Our data, however, preclude definite inferences in this area.

PSS is a rare event but nonetheless a major concern in asplenic patients [12], [21]. Although registry studies place the rate of PSS at 13/1000 person years of observation (PYO) [19], we were unable to detect an event of definite PSS in the intervention group of our cohort after three years of follow up (equivalent to 237 PYO). This might in part be due to the higher rate of preventive measures in our cohort. Importantly, though, we were unable to determine the cause of death in seven patients and an additional nine patients did not respond at all, which may have led to underdetection of PSS events.

Since this cohort was heavily influenced by the COVID-19 pandemic we sought to analyze the COVID-19-related outcomes of the participants. Although recommendations concerning vaccinations against COVID-19 were not incorporated in our intervention, the vast majority of participants received multiple vaccinations against SARS-CoV2. Somewhat surprisingly, the number of PCR-confirmed cases in our cohort is quite low but there is possibly some underreporting present due to decreased availability of PCR-tests in the later stages of the pandemic. Our study was not designed to allow for estimates of incidence of COVID-19 among asplenic patients, nevertheless we deem it noteworthy that there was only one case of severe COVID-19, which occurred before the widespread availability of vaccinations. Data regarding the outcome of COVID-19 in asplenic patients are still scarce and date either from very early in the pandemic [23] or rely mainly on self-reported health data [4]. Given the comparatively low incidence and benign clinical courses of COVID-19 observed in our cohort, however, our data do not indicate a propensity for severe COVID-19 among asplenic patients.

Our study has some limitations. While we tried to ascertain the status and, where applicable, cause of death of every participant by contacting general practitioners and next of kin, in some patients these questions could not be answered. Thus, our data may underestimate the occurrence of PSS in this cohort. However, in many cases which were lost to follow up, malignoma was already established at inclusion and seems a likely candidate for the reason of the loss to follow up. Additionally, due to the non-randomized design of our study we cannot rule out residual confounding. Although we used a well –designed statistical model to account for this, unmeasured confounding might still be present.

Our study also has several strengths. The use of the Health Action Process Approach (HAPA) theory for the intervention is innovative and emphasizes patient empowerment and motivation, which are critical for sustained behavioral change. The study’s design, incorporating a long-term follow-up of three years and the comparatively high follow-up completion rate provide robust insights into the durability of the intervention’s effects.

In conclusion, patients who had received our novel HAPA-based telephonic intervention exhibited significant and sustained improvement in adherence to guideline-based preventive measures. By emphasizing patient empowerment, our intervention may serve as a template for other preventive efforts aiming to increase long-term adherence to standard of care.

## Data Availability

No datasets were generated or analysed during the current study.
